# Reproducibility and Consistency of Proteomic Experiments on Natural Populations of a Non-Model Aquatic Insect

**DOI:** 10.1371/journal.pone.0104734

**Published:** 2014-08-18

**Authors:** Amparo Hidalgo-Galiana, Marta Monge, David G. Biron, Francesc Canals, Ignacio Ribera, Alexandra Cieslak

**Affiliations:** 1 Animal Biodiversity and Evolution program, Institute of Evolutionary Biology (CSIC-Universitat Pompeu Fabra), Barcelona, Spain; 2 Vall d'Hebron Institute of Oncology (VHIO) Edifici Collserola, Barcelona, Spain; 3 Laboratoire “Microorganismes: Génome et Environnement”, UMR CNRS 6023, Equipe Interactions hôtes-parasites, Université Blaise Pascal, Aubière, France; University of Hong Kong, Hong Kong

## Abstract

Population proteomics has a great potential to address evolutionary and ecological questions, but its use in wild populations of non-model organisms is hampered by uncontrolled sources of variation. Here we compare the response to temperature extremes of two geographically distant populations of a diving beetle species (*Agabus ramblae*) using 2-D DIGE. After one week of acclimation in the laboratory under standard conditions, a third of the specimens of each population were placed at either 4 or 27°C for 12 h, with another third left as a control. We then compared the protein expression level of three replicated samples of 2–3 specimens for each treatment. Within each population, variation between replicated samples of the same treatment was always lower than variation between treatments, except for some control samples that retained a wider range of expression levels. The two populations had a similar response, without significant differences in the number of protein spots over- or under-expressed in the pairwise comparisons between treatments. We identified exemplary proteins among those differently expressed between treatments, which proved to be proteins known to be related to thermal response or stress. Overall, our results indicate that specimens collected in the wild are suitable for proteomic analyses, as the additional sources of variation were not enough to mask the consistency and reproducibility of the response to the temperature treatments.

## Introduction

The comparison of natural populations using proteomic methods, which has been termed “population proteomics” [1], [2] has a high potential to address fundamental questions in ecology and evolutionary biology, as it allows us to directly link environmental conditions to changes in protein expression [3]–[6]. Proteomic methods are especially suited to understanding phenotypic changes induced by the environment, since they enable detection of alterations affecting physiologically significant protein expression and modification, rather than changes in mRNA expression levels [7]–[9]. When applied to different populations exposed to varying environmental conditions, differences in proteome expression are likely to be directly linked to a physiological response [1], [10], [11].

Although proteomic studies of non-model organisms are increasingly common (see e.g. [8] for review), in most cases specimens are kept in the laboratory under controlled conditions, as the use of specimens directly taken from their natural environment poses an additional challenge [12]. Many unknown factors, such as the genetic background of the individuals, their age, the physiological state, the presence of parasites or other pathogens may introduce variation of unpredictable importance [13]–[15]. Thus, it seems that previous to any comparative study of wild populations it is necessary to estimate the degree of variability due to unknown or unforeseeable sources of variation, and to assess both the reproducibility and consistency of the protein expression data.

In the study presented here we approach this problem using two natural populations of a diving beetle species (*Agabus ramblae* Millán & Ribera). This species has a disjunct distribution in the South and East of the Iberian Peninsula and Central Morocco, and is usually found in highly mineralized, temporary running waters [16]. It belongs to a complex of three closely related species (the *Agabus brunneus* group [16]) distributed in the western Mediterranean, which most likely diversified during the Pleistocene, within the Iberian peninsula [17]. Variation in protein expression was quantitatively assessed with 2-D Differential Gel Electrophoresis (2D-DIGE). The experimental setup also included investigation of temperature induced protein expression, as temperature is one of the most important abiotic factors known to influence a wide range of physiological reactions [18]–[20]. In the evolutionary lineage the studied species belongs to, thermal tolerance is known to be related to the size of the geographical range [21]. In the context of a wider study of ecological segregation and speciation in *A. ramblae* and its most closely related species, we studied the response of two geographically separated populations (in Central Morocco and Southern Spain) to two temperatures at the extremes of what they may experience in the field. Our main interest was to assess the possibility of comparing the overall protein expression of wild populations subjected to different temperature treatments, without being overwhelmed by confounding variation. We specifically aimed to determine the variability of 1) technical replicas, by comparing the internal standards of each experiment; 2) biological replicas, by comparing pooled samples of several individuals of the same population exposed to the same treatment (referred to as “replicated samples”); 3) temperature treatment, by comparing the different treatments within the same population; and 4) the response of the specimens of two different populations to the same treatment.

## Methodology

### Studied populations, acclimation

In order to determine potential differences in response to temperature treatment, two natural populations of the diving beetle *Agabus ramblae* (Coleoptera, Dytiscidae, size range 7–9 mm) were used for the experiments: 1) Spain, Murcia, Corneros stream N37°42′10.7″ W1°55′33.8″ (30 adult specimens); and 2) Morocco, Tinghir, Toudgha river N31°33′25.1″ W5°34′49.5″ (24 adult specimens). *Agabus ramblae* is not included in any national or international list of protected or endangered species, and the two populations were in public land not covered by any special legal protection. No permits or ethical approval were required for the experimental procedures. The two populations were sampled in September 2007 and May 2011 respectively, and all specimens that could be found during a search of 2–3 h in the available microhabitats were collected and transported under similar conditions to the laboratory. Three specimens of the Spanish population were snap frozen in the field in liquid nitrogen, serving as a field control (FC).

Once in the laboratory, individuals were acclimated for one week in aquaria, with mineral water and some vegetation taken from the place of origin. Specimens were kept at room temperature (RT, always below 25°C), which was considered the control for these experiments, and with a natural day/night cycle.

Specimens were fed *ad-libitum* on frozen red Chironomidae larva from commercial sources (sold as fish food). After a week, an equal number of specimens were randomly allocated to each of three treatments for 12 h: 4°C, RT, and 27°C. This is within the range of temperatures the species are likely to experience under natural conditions (WorldClim_2.5 m database). After the treatment, specimens were snap frozen in liquid nitrogen, separated into three samples of 2–3 specimens for each temperature treatment and stored at −80°C. The number of specimens per replicated sample was limited by the total number of specimens available and the need of having at least three replicas per treatment for comparison. By using only 2–3 specimens we increased the potential variability between replicated samples, so it can be expected that by using more specimens this individual variability could be further reduced.

### Protein extraction and sample preparation

Proteins of whole specimens were extracted in a solution of 9.5 M urea, 1% Dithiothreitol (DTT), 2% (3-cholamidopropyl)-dimethyl-ammonia (CHAPS) and 2% Pharmalyte™ (pH gradient 3–11), using a mortar and liquid nitrogen to maintain the low temperature [22]. The samples were sonicated after extraction to break up nucleic acids. After centrifugation at 13,200 rpm for 2 min the supernatant was transferred into a new tube for further processing. Samples were precipitated using 2-D-CleanUp kit (GE/Amersham Biosciences, Freiburg, Germany) to remove interfering contaminants. The total protein was then resuspended in an appropriate volume of DIGE lysis buffer (Tris 30 mM, Urea 7 M, Tiourea 2 M, CHAPS 4%, HCl to reach pH 8.5). Samples were quantified with a Bio-Rad RCDC Protein Assay (Bio-Rad, Hercules, CA, USA).

### Experiment design and DIGE analysis

An internal standard for each experiment was generated by pooling equal amounts of protein from each extraction. Five gels per experiment were run with the internal standard and two samples derived from different treatments.

Sample aliquots of 50 µg were labelled with Cy3 and Cy5 NHS ester and the pooled internal standard was labelled with Cy2 (GE Healthcare, Buckinghamshire, UK), according to the Ettan DIGE minimal labelling protocol (GE Healthcare). To avoid any possible bias due to labelling efficiency, the samples of each group were alternately labelled with both Cy3 and Cy5 dyes. The DIGE experiments followed the standard protocol as described in ref. [23]. Gel images were obtained with a Typhoon 9400 scanner (GE Healthcare). Images were scanned at 550/580, 560/620 and 525/555 nm excitation/emission wavelengths for the Cy2, Cy3 and Cy5 dies respectively, at 100 µm resolution. 2D-DIGE image analysis and statistical quantification of relative protein abundance were performed with Progenesis SameSpots v4.0 (Nonlinear Dynamics, Newcastle, UK). This software allows detecting, quantifying and matching of spots between gels after normalization to the internal standard. Statistically significant differences in protein expression between groups (temperature treatments) were tested with one-way ANOVA. To correct for multiple tests we used the false discovery rate correction (FDR) as implemented in SameSpots (*q*<0.05).

### Statistical analyses

Gel images were aligned with reference to the internal standard, normalized, and the protein spots verified. For each experiment, pairwise comparisons of the expression level between replicated samples and between treatments were done, selecting those spots which indicated a significant difference according to the ANOVA analysis at *P*<0.05, *P*<0.01 and *P*<0.001 levels. The analyses included: 1) experimental variation due to technical error; 2) variation between replicated samples, in order to detect variability due to individual differences (genetic background, sex, age, physiological state); 3) variation between treatments within the same population; and 4) variation between treatments across populations. To determine the variation due to technical reasons, for each experiment the five images of the internal standard were compared using the ‘single stain per gel’ option in SameSpots software to detect false positives. We also computed the coefficient of variation (CV) among the spot volumes across the different replicas, averaged for all spots (CV = SD/mean ×100; [24]).

To assess the variability between replicated samples or treatments within the same population we calculated the distribution of the differences in expression of the same protein spot for each comparison, generating a matrix of protein expression data which showed significantly different level of expression at the selected *P*-level. With these matrixes we did a hierarchical cluster analysis using the Euclidean distance and Ward's amalgamation method (see [1] for comparison). We used the single available field control to have an estimate of the changes introduced by the acclimation process, with a standardised food supply.

For a global comparison of treatments across populations we performed an ANOVA analysis for those protein spots exhibiting significant differences of expression level in the pairwise comparison between treatments within the same population. We considered *P*-level, temperature treatment and population as factors. All statistical analyses were done with Statistica version 7 (http://www.statsoft.com) and JMP v5.1 [25].

### Protein selection and identification

#### Selection of protein spots

We selected and identified protein spots with different levels of expression between experiments as a proof of principle, to test the viability of our approach and methodology for the study of the thermal biology of *Agabus ramblae*. For this purpose, we set up a new analysis in SameSpots v4.0 by directly comparing all images. All proteins spots were first automatically selected and then manually checked for consistency and quality of image, with a final selection of 565 protein spots common to all experiments. We then compared the expression level of the 565 protein spots in the 27°C *vs* 4°C replicas, irrespective of the population of origin, as these were the ones most likely to show strong differences in protein expression. We used the normalized spot volumes to estimate fold changes, and compared the values for each spot using a one-way ANOVA with a cut-off absolute value of>1.3-fold (*P*<0.05, with FDR correction). The normalized volume of the protein spots with significant differences in expression was used in a Multiple Discriminant Analysis (MDA) to identify the protein spots that better discriminate between treatments. Finally, the selected protein spots were double-checked again in SameSpots v4.0, where the final selection was made.

#### Protein identification and Liquid chromatography-Mass spectrometric analysis

A new preparative gel was run to extract and identify target proteins. Three hundred micrograms of a mix of protein extracts from representative samples were Cy labelled and gels were scanned and images analysed as described above. The gel images were matched against the spots referenced in the picking list created after the detection of the significantly up- or down-regulated protein signals in the gels used for the analyses. The selected protein spots were excised from the gel using an automated Spot Picker (GE Healthcare), within-gel digestion with trypsin (Promega, Wisconsin, USA) as described in [26].

Extracted samples were analysed on a Maxis high resolution Q-TOF spectrometer (Bruker, Bremen), coupled to a nano-HPLC system (Proxeon, Denmark). After evaporation and dissolution in 5% acetonitrile 0.1% formic acid in water, samples were first concentrated on a 100 µm ID, 2 cm Proxeon nanotrapping column and then loaded onto a 75 µm ID, 15 cm Acclaim PepMap nanoseparation column (Dionex). Chromatography was run using a 0.1% formic acid - acetonitrile gradient (5–35% in 10 min; flow rate 300 nL/min). The column was coupled to the mass spectrometer inlet through a Captive Spray (Bruker) ionization source. MS acquisition was set to cycles of MS (1 Hz), followed by MS/MS (0.5–2 Hz) of the 8 most intense precursor ions with an intensity threshold for fragmentation of 5000 counts and using a dynamic exclusion time of 0.5 min. All spectra were acquired on the range 100–2200 Da. LC-MSMS data was analysed using the Data Analysis 4.0 software (Bruker).

Proteins were identified using Mascot (Matrix Science, London UK) by search on the NCBI database limiting the search to Other Metazoa (13,577,271 sequences; 4,662,347,403 residues). MS/MS spectra were searched with a precursor mass tolerance of 10 ppm, fragment tolerance of 0.04 Da, trypsin specificity with a maximum of 2 missed cleavages, cysteine carbamidomethylation set as fixed modification and methionine oxidation as variable modification.

## Results and Discussion

Specimens of *A. ramblae* weighed between 35–75 mg. The average amount of total protein per specimen was ca. 1500 µg, with no significant differences between populations (2-tail t-test, *P*>0.1; Table 1).

**Table 1:** Protein yield obtained from each replicated sample.

population	Replicated sample	ind./replica	average prot(µg)/ind
SE Spain (Murcia)	FC	3	498,7
	RT_r1	3	603,6
	RT_r2	3	1343,4
	RT_r3	3	1362,8
	4_r1	3	1113,7
	4_r2	3	1376,7
	4_r3	3	1184,5
	27_r1	3	1130,6
	27_r2	3	1380,7
	27_r3	3	1045,5
C Morocco (Tinghir)	RT_r1	2	3829.3
	RT_r2	2	448.4
	RT_r3	2	1186.1
	4_r1	2	1193.4
	4_r2	2	1400.2
	4_r3	2	1197.9
	27_r1	2	2503.3
	27_r2	2	2969.5
	27_r3	2	1551.7

### Technical variation

The five images of the internal standards were compared in both populations to detect the technical variation of the experimental setup using the single stain option. When the images were grouped in different combinations, the number of protein spots with significantly different levels of expression at a statistical threshold of *P*<0.05 ranged between 2–4% (Table 2). This rate of false positives could be considered as technical error in our experimental setup. The technical variation estimated by comparison of the internal standards is in fact an overestimation, as it is not corrected by the normalization. The maximum value of the coefficient of variation (CV) between technical replicas was 35%, within the standard range for 2-DE experiments (20–40%, [27]).

**Table 2:** Comparison between the five internal standards within each experiment (population) to estimate technical variation.

population	ANOVA	Spots signif. diff. *P*<0.05	max	min	avrg
SE Spain	12*vs*345	97	13.7	1.1	1.9
(Murcia)	14*vs*235	49	3.9	1.1	1.8
	135*vs*24	95	4.0	1.1	1.8
C Morocco	12*vs*345	56	2.6	1.1	1.4
(Tinghir)	14*vs*235	48	2.6	1.1	1.5
	135*vs*24	46	2.1	1.1	1.4

ANOVA, different groupings of the internal standards for the ANOVA test (see main text). Values, fold change.

Reverse labelling was used to minimize any possible bias due to preferential labelling with one of the Cy dyes. When images of samples belonging to the same group and labelled alternatively with Cy3 or Cy5 were compared, no significant differences were observed. This behaviour is consistent with previous reports that have shown that labelling is only a very minor source of variability in DIGE experiments [28].

### Variation between replicated samples

Differences between individual histories and circumstances are one of the major sources of variation in expressed physiological traits [29]. We tried to minimize this variation by including several specimens per replica [30], [31], as we wanted to assess population-level, not individual responses to thermal stress or other environmental factors. As noted in the methods, the final number of specimens per replicated sample was a trade-off between the availability of specimens and the need for replicas in the 2-DE experiments.

**Figure 1:**
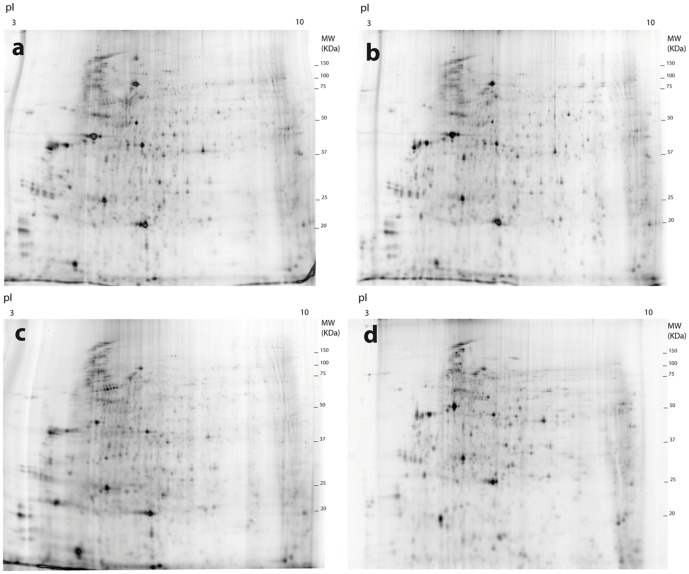
Example images of 2D-DIGE gels representing populations and temperature treatments. a) Spanish population, 4°C treatment, Cy5; b) Spanish population, 4°C treatment, Cy3; c) Spanish population, 27°C treatment, Cy3 and d) Moroccan population, 27°C treatment, Cy3. Differences between a) and b) correspond to variation between replicated samples; a) and c) different treatments within the same population; and c) and d) same treatment between different populations. pI, isoelectric point; MW molecular weight (Kilo Daltons).

**Figure 2:**
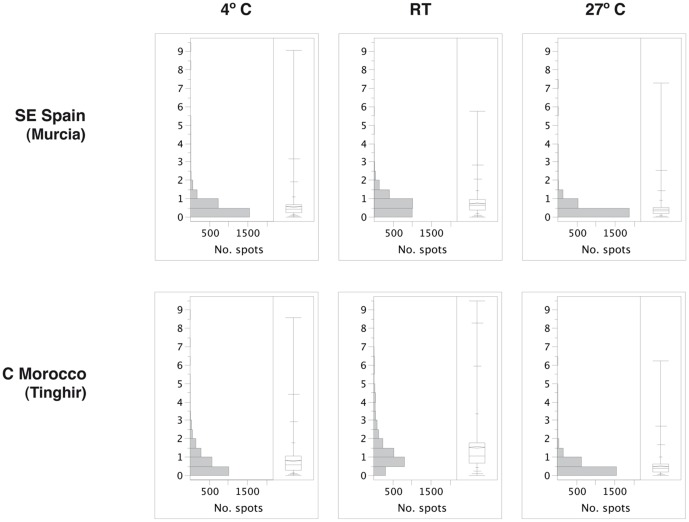
Distribution of differences between the three replicated samples of each treatment. Data reflect normalised protein spot volume. On the right of each graph the quantile box plot reflects the distribution of the variation, with mean (rhomboid) and median. Vertical axis, fold change.

The distribution of the differences in protein expression between the replicated samples of the same treatment were very similar in the two populations (Figures 1, 2). Among the samples of the 4° and 27°C treatments more than 50% of the protein spots show differences of expression levels of less than 0.5 fold change, although the replicas at RT had a higher overall variation, with the median between 0.5–1 fold for the Moroccan population (Figure 2, Tables S1, S2). The CV among replicated samples ranged between 40–60% for the 4°C and 27°C treatments, and between 75–126% for the replicas at RT, higher than the variation between technical replicas (see above) [32] but similar to other reported measures of biological variation [33]. RT samples were exposed to fluctuating temperatures within a range that can be considered normal for the species, and therefore the spectrum of expressed proteins can be expected to be wider than that of specimens exposed to a constant extreme temperature, with a more selective protein expression.

A potentially important factor may be the existence of cohorts in the studied population, which could reduce the inter-individual variability of specimens collected at the same time in the same area, but may show increased variability throughout the year or between different geographical areas. The life cycle of *Agabus ramblae* is not known in detail [32], and therefore it is not possible to predict the population structure at any given time. Although larvae are more often reported from March to June [34], adults can be found any time of the year, as usual for lowland species in the Southern part of the Iberian Peninsula and Morocco. So it seems likely that a mixture of adults of different origin, age, gender and physiological state were included in the samples. In any case, it has been shown that males and females of the same species had identical values for upper and lower thermal limits [21].

### Intra-population analysis on the effect of temperature treatments

**Figure 3:**
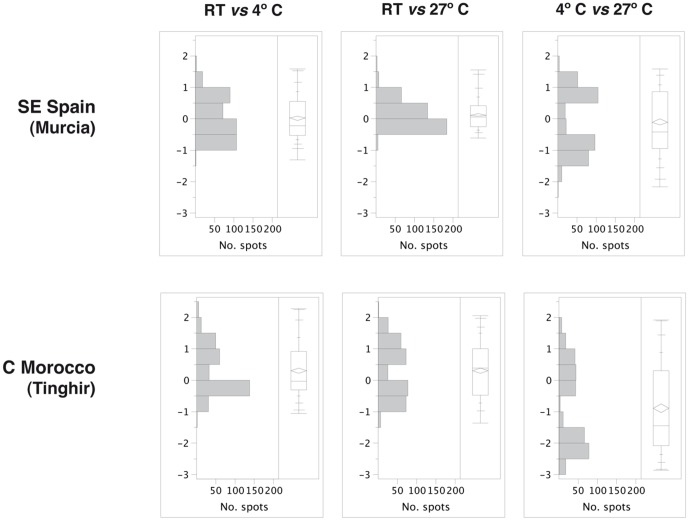
Distribution of pairwise differences in protein spot volume between temperature treatments in each population. On the right, distributions in a quantile box plot, including mean (rhomboid) and median. Vertical axis, fold change.

Around 40% of all the protein spots had significantly different expression levels between treatments at *P*<0.05 (Table 3). The largest differences were detected between the two extreme temperature treatments (4°C and 27°C). Of all the protein spots with significant differences at *P*<0.05, between 95–99% were different between these two treatments in both populations (Table 3). The false discovery rate correction (FDR) reduced the number of spots with significant differences by 2.6% and 8.1% for the south Spanish and Moroccan populations respectively, but did not change the overall pattern. The distribution of the pairwise differences between treatments was in general bimodal and approximately symmetrical, especially for the comparisons between the 4 and 27°C treatments (Figure 3, Tables S1, S2). Only for the comparison between RT and 27°C of the Spanish population the distribution was unimodal, with modal values between 0 and 0.5 fold change (i.e. most protein spots showed little or no differences) (Figure 3, Tables S1, S2). The range of variation was similar for all comparisons, with most spots between +/−2 fold change, with generally higher values for the comparison between 4 and 27°C.

**Table 3:** Number of protein spots with a significantly different level of expression.

Population	*P* level	all PS.	comparison	PS	fold>1.5
SE Spain (Murcia)	<0.05	856	RT *vs* 4°C	81	64
			RT *vs* 27°C	46	35
			4°C *vs* 27°C	811	514
			4°C *vs* RT *vs* 27°C	467	291
	<0.01	402	RT *vs* 4°C	15	11
			RT *vs* 27°C	4	0
			4°C *vs* 27°C	385	267
			4°C *vs* RT *vs* 27°C	84	66
	<0.001	79	RT *vs* 4°C	0	0
			RT *vs* 27°C	0	0
			4°C *vs* 27°C	77	62
			4°C *vs* RT *vs* 27°C	5	5
C Morocco (Tinghir)	<0.05	755	RT *vs* 4°C	63	62
			RT *vs* 27°C	63	61
			4°C *vs* 27°C	716	715
			4°C *vs* RT *vs* 27°C	334	333
	<0.01	451	RT *vs* 4°C	4	4
			RT *vs* 27°C	2	2
			4°C *vs* 27°C	439	439
			4°C *vs* RT *vs* 27°C	96	96
	<0.001	120	RT *vs* 4°C	0	0
			RT *vs* 27°C	0	0
			4°C *vs* 27°C	119	119
			4°C *vs* RT *vs* 27°C	6	6

All PS, overall number of protein spots with significant differences; PS, number of protein spots with significant differences in the pairwise comparison between treatments; fold >1.5, number of protein spots with fold differences above 1.5.

**Figure 4:**
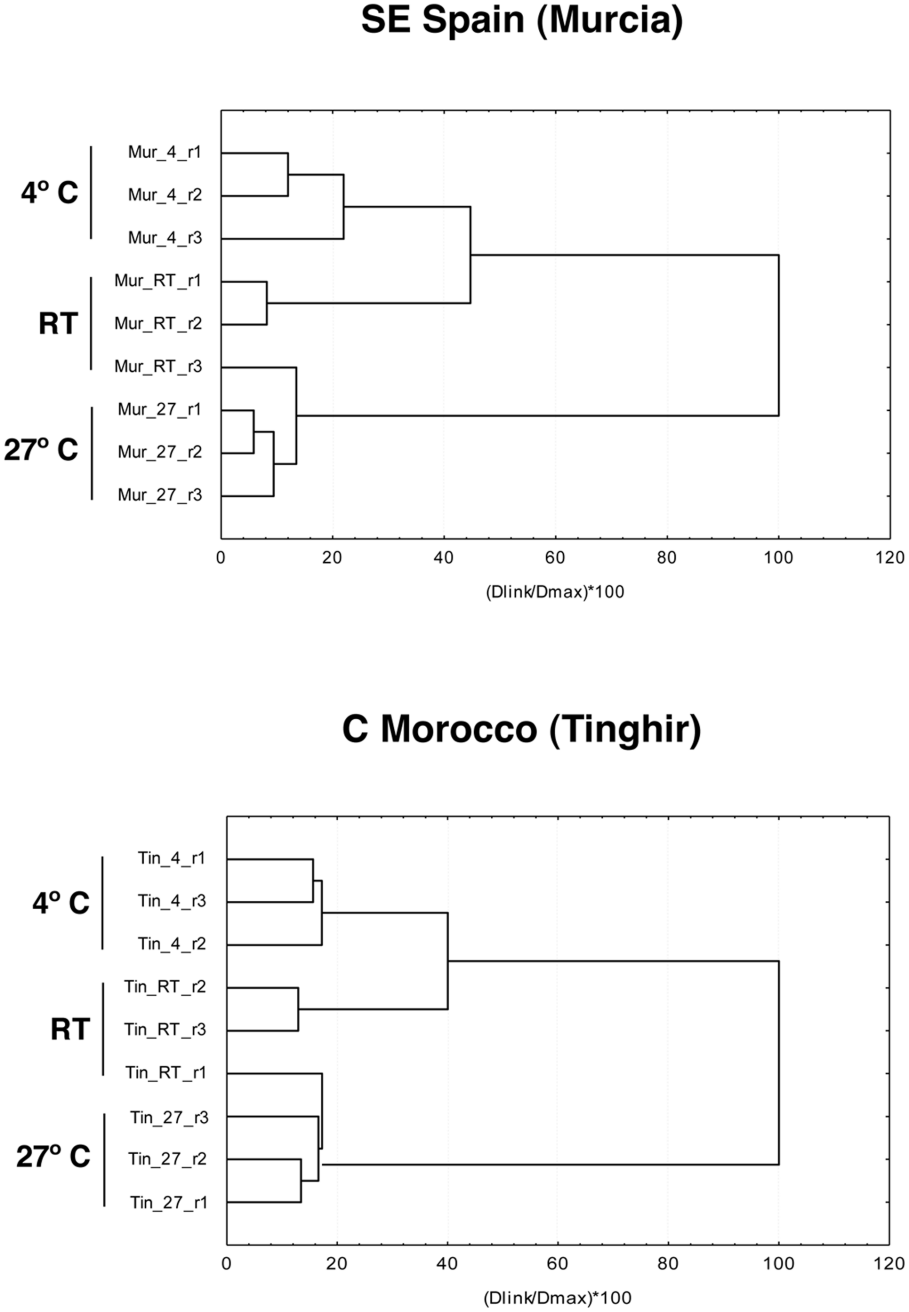
Cluster analysis of the number of significantly differently expressed protein spots. The analysis includes the proteins with significantly different expression for each replicated sample, as measured with ANOVA at *P*<0.01 (see Tables 3, 4). r1 to r3, replicated samples.

In the hierarchical cluster analysis of the two populations, the protein spots significantly different in expression at both *P*<0.05 and *P*<0.01 resulted in a clear grouping of the biological replicas of the 4°C and 27°C treatments, with these two treatments showing the split at the deepest level. Differences within treatments were minimal relative to differences between treatments (Figures 1, 3, Figure S1). However, the replicas of RT were inconsistently clustered with the 4°C or the 27°C treatments. At the *P*<0.05 level some RT replicas were nested within the treatments (Figure S1), but at *P*<0.01 level the replicas of the two treatments were clustered together to the exclusion of the RT replicas (Figure 4). At P<0.001 the number of protein spots with significant differences was not high enough for a meaningful cluster analyses (Table 3). As already noted, in the RT treatment specimens were not subjected to a particular stress factor after their acclimation period, therefore their protein expression may represent a basic metabolic state with no compensatory reaction, with a wider range of intra-sample variation. In contrast, specimens subjected to extreme temperature treatments (4° and 27°C) reflected the influence of these stressful conditions by a stereotype modification of the protein expression pattern, with ca. 30% of the total number of protein spots significantly varying between these two treatments at *P*<0.05 level (Table 3).

The effect of the transport and acclimation period in the laboratory previous to the experiments and protein extraction could also have resulted in an artificially higher homogenisation of intra-experimental variability. All specimens were kept under the same conditions and fed on a homogeneous diet during one week, potentially reducing variation due to their individual history (starvation, consumption of different preys). This homogeneity in the experimental conditions could have introduced an artefact by modifying the protein expression in a similar way in all specimens. That this was not the case, and that the influence of the transport and acclimation was not reflected in the protein expression pattern, could be shown by the analysis of the field control of the Spanish population. The high overlap in the pairwise comparison of the RT-samples and the field control (Figure S2) indicates that neither the basic metabolism changed significantly nor the uniform food and conditions resulted in a higher homogeneity, although, due to the difficulty in obtaining enough specimens, only one replicated sample of a field control could be studied.

### Comparison of the same treatment between populations

The number of protein spots that showed significant differences in the pairwise comparison of treatments was very similar for the two populations at each of the *P* levels used (Table 3, Figure 1). Differences between populations were not significant (as measured with ANOVA, *P*>0.8, Table 2), while differences for treatments and for the *P*-levels used for selecting the protein spots included in the comparison were highly significant (*P*<0.001, Table 4).

**Table 4:** Results of the ANOVA comparison between populations.

Factor	DF	Sum of squares	F Ratio	Sig.
population	1	737	0.05	>0.8
*P* level	2	363344	11.10	<0.0001
treatment	3	644782	13.13	<0.001

See Table 3 for the number of protein spots with significant differences between treatments at each *P* level.

Despite the geographical distance, the general climatic conditions between Murcia and Tinghir are similar: annual average maximum temperatures are 30.8°C and 37.0°C respectively, and minimum temperatures 2.1°C and 1.2°C (WorldClim 2.5 m database, [35]). Monthly average maximum and minimum temperatures, for the month in which the specimens were collected, are 27.0°C and 14.8°C for Murcia (September) and 30.0°C and 15.2°C for Tinghir (May). General climatic conditions may however not reflect the particular thermal circumstances which the species are exposed to [36], especially for freshwater organisms living submerged [37]. In order to identify potential physiological reaction norms in either of the populations, preliminary data on the thermal tolerance of the Moroccan population were obtained by the same methodology as described in [21]. The average UTL (Upper Thermal Limit) for the Spanish population was 45°C [21], while the Moroccan population only reached 43.6°C. The average LTL (Lower Thermal Limit) of the Moroccan population was −6.8°C, identical to the LTL reported for the Spanish population [21]. There thus seems to be only slight differences between the thermal tolerances of both populations, which were reflected in the similar results of our comparative study of their response to different temperatures.

### Protein identification

We selected 10 spots with significantly different expression levels between temperature treatments as measured with ANOVA (*P*<0.05 with FDR correction, cut-off values of >1.3 fold), and with the highest discriminant values in the MDA. Of these, three protein spots were selected for a preliminary analysis. These were identified as a chaperone (heatshock cognate Hsc70), a structural protein (alpha actinin) and a protein involved in the energy metabolism and membrane ion transport (sarco(endo)plasmic reticulum-type calcium ATPase) (Table 5).

**Table 5:** Exemplary identified proteins.

no.spot Protein	2180 Hsc70	1727 alpha actinin	483 sarco(endo)plasmic reticulum-type calcium ATPase
regulation	up-regulated at 27°C	up-regulated at 27°C	down-regulated at 4°C
fold change	3.12	8.5	5.8
sequence	DAGTIAGLNVMR	QTDNSLAGVQK	VGEATETALIVLAEK
score	52	67	85
IP	5.02	5.60	5.32
MW	82	100	64
Acc.No.	AB122065	NP_726784	AF115572
functional category	Chaperone	structural	energy, transport
organism	*Crassostrea gigas*	*Drosophila melanogaster*	*Heliconius virescens*

Included are the amino-acid sequence of the identified fragment with its isoelectric point (IP), molecular weight (MW) and the score of the search in the database, the identification, and the NCBI accession number and organism of the best match.

#### Hsc70

This protein was up-regulated at 27°C in both populations. The same effect has been reported for the same or related proteins in several groups of animals (Heteroptera [38], Tunicata [39], leaf beetles [40]), although there are also reports of up-regulation at low temperatures (e.g. [41]). It belongs to Hsp70 family, regulating the ATP-dependent folding of proteins [41]. The expression of proteins of the Hsp70 family might be up-regulated as a response to temperatures routinely experienced in nature, and is related to thermal tolerance. It is considered to be a much more sensitive and ecologically relevant indicator of sub-lethal thermal stress, hence important in establishing the limits of the distribution of species or populations along environmental temperature gradients [40]. Experiments using RNAi to suppress Hsp70 translation prevented completely the recovery from heat shock, and also affected negatively the repair of chilling injury in insects [38].

#### Alpha actinin

This protein was also up-regulated at 27°C, as reported in other studies (e.g. [39]). The alpha actinins belong to the spectrin gene super-family that represents a diverse group of cytoskeletal proteins. Alpha actinin is an actin-binding protein with multiple roles in different cell types.

#### Sarco(endo)plasmic reticulum-type calcium ATPase (SERCA)

The SERCA protein was found to be down-regulated at 4 °C. It is a protein involved in removing calcium from the cytoplasm to maintain the low concentration necessary for cell signalling, known to be temperature dependent in insects [42]–[43]. The differences in expression in *Agabus ramblae* may suggest that this species may show cold hardening, something that would require experimental data to confirm.

### Concluding remarks

In this work we show that it is possible to conduct proteomic studies on wild populations of non-model organisms to obtain physiologically relevant data with relatively less noise. The reproducibility and uniformity of the results presented here for two distinct populations of a species of water beetle (*Agabus ramblae*) suggest that the experimental setup allowed the detection of a common stress-related response to temperatures at the extremes of the range they experience in their natural environment. We selected and identified some example proteins, and found that, in agreement with previous work [39], [41], up-regulated proteins at higher temperatures were involved in structural protection, and down-regulated proteins at low temperatures in the reduction of metabolic activity and energy expenditure. Our work opens the possibility of a wider use of comparative population proteomics in wild populations of non-model organisms, with a vast potential to address a whole range of basic questions in ecology and evolutionary biology. The use of wild populations not only allows the study of species for which common garden experiments are not feasible, but also the study of the interaction with local conditions. If differences in the reaction norm of local populations were due to environmental imprinting, common garden experiments may mask phenotypic variability that could be potentially important to explain evolutionary processes at the edge of the geographical range of species [44]–[46].

## Supporting Information

Figure S1 **Cluster analysis of the number of significantly differently expressed protein spots (** ***P*** **<0.05).** The analysis includes the proteins with significantly different expression for each replicated sample, as measured with ANOVA at *P*<0.05 (see Tables 3, 4).
(EPS)

Figure S2 **Cluster analysis of the number of significantly differently expressed protein spots including the field control (FC).** The analysis includes the proteins with significantly different expression for each replicated sample plus the field control (FC) as measured with ANOVA at *P*<0.01 (see Tables 3, 4) of the Spanish population.
(EPS)

Table S1 **Normalised volume of the spots detected in the Spanish population.** Included, whether the spot had significant differences for any of the ANOVA comparisons and was included in the subsequent analyses (Yes) or not (No) (all spots were included in the histogram in Fig. 2); SPA (Spain); 4, RT and 27, temperature treatments (4°C, room temperature and 27°C respectively); 1, 2 and 3, biological replicas.
(XLSX)

Table S2 **Normalised volume of the spots detected in the Moroccan population.** Included, whether the spot had significant differences for any of the ANOVA comparisons and was included in the subsequent analyses (Yes) or not (No) (all spots were included in the histogram in Fig. 2); MOR (Morocco); 4, RT and 27, temperature treatments (4°C, room temperature and 27°C respectively); 1, 2 and 3, biological replicas.
(XLSX)
